# Reliable classification of facial phenotypic variation in craniofacial microsomia: a comparison of physical exam and photographs

**DOI:** 10.1186/s13005-016-0109-x

**Published:** 2016-03-31

**Authors:** Craig B. Birgfeld, Carrie L. Heike, Babette S. Saltzman, Brian G. Leroux, Kelly N. Evans, Daniela V. Luquetti

**Affiliations:** Division of Plastic Surgery, Department of Surgery, University of Washington, Seattle, WA USA; Craniofacial Center, Seattle Children’s Hospital, Seattle, WA USA; Center for Clinical and Translational Research, Seattle Children’s Research Institute, Seattle, WA USA; Department of Pediatrics, University of Washington, M/S OB.9.520, PO Box 5371 4800 Sand Point Way, Seattle, WA 98105 USA; Department of Biostatistics, School of Public Health and Oral Health Sciences, School of Dentistry, University of Washington, Seattle, WA USA; Center for Developmental Biology and Regenerative Medicine, Seattle Children’s Research Institute, Seattle, WA USA

**Keywords:** Reliability, Craniofacial microsomia, Hemifacial microsomia, PAT-CFM, Photographs, Physical exam, Multicenter, Clinical research, Craniofacial, Image Protocol

## Abstract

**Background:**

Craniofacial microsomia is a common congenital condition for which children receive longitudinal, multidisciplinary team care. However, little is known about the etiology of craniofacial microsomia and few outcome studies have been published. In order to facilitate large, multicenter studies in craniofacial microsomia, we assessed the reliability of phenotypic classification based on photographs by comparison with direct physical examination.

**Methods:**

Thirty-nine children with craniofacial microsomia underwent a physical examination and photographs according to a standardized protocol. Three clinicians completed ratings during the physical examination and, at least a month later, using respective photographs for each participant. We used descriptive statistics for participant characteristics and intraclass correlation coefficients (ICCs) to assess reliability.

**Results:**

The agreement between ratings on photographs and physical exam was greater than 80 % for all 15 categories included in the analysis. The ICC estimates were higher than 0.6 for most features. Features with the highest ICC included: presence of epibulbar dermoids, ear abnormalities, and colobomas (ICC 0.85, 0.81, and 0.80, respectively). Orbital size, presence of pits, tongue abnormalities, and strabismus had the lowest ICC, values (0.17 or less). There was not a strong tendency for either type of rating, physical exam or photograph, to be more likely to designate a feature as abnormal. The agreement between photographs and physical exam regarding the presence of a prior surgery was greater than 90 % for most features.

**Conclusions:**

Our results suggest that categorization of facial phenotype in children with CFM based on photographs is reliable relative to physical examination for most facial features.

## Background

Craniofacial microsomia (CFM) is a congenital condition occurring in 1 in 3000 to 1 in 5000 live births [1, 2] and it is the second most common congenital facial condition after cleft lip and palate [2–4]. CFM variably affects derivatives of the first and second pharyngeal arches [5, 6] thereby creating a wide spectrum of phenotypic severity. Established diagnostic criteria for CFM do not exist, and the etiology of CFM is unknown for most patients. The combination of a lack of knowledge about the etiology and the wide variability in clinical presentation of CFM has hampered our ability to evaluate immediate and long term treatment outcomes; thus, we lack sufficient evidence to establish standardized treatment protocols. The published literature on CFM has primarily been limited to clinical reviews, case series, and reports of clinical classification systems. Due to the heterogeneous nature of this condition, multicenter studies are required for sufficiently powered analyses comparing treatment outcomes within phenotypic subgroups.

There are numerous inherent challenges in conducting a multicenter research study in CFM, not least of which is the need to establish a standardized phenotypic assessment of study participants. An accurate assessment of craniofacial features is imperative for studies reliant on meaningful comparisons among individuals with CFM. Historically, a direct physical exam has been considered the gold standard for such an appraisal. Performing reliable direct physical examinations can be challenging by virtue of the distances between study sites, variation in classification between clinicians, challenges inherent in completing a thorough exam in person (such as impact on child and ability to dedicate/coordinate time between participant and qualified rater). However, photographs are relatively easy to obtain, are often included in clinical visits, and have been incorporated into studies in similar craniofacial conditions [7–9]. Therefore, we sought to compare assessments on photographs and physical examination.

Classification systems such as Pruzansky [10], SAT [11], OMENS [12], and OMENS+ [13] can facilitate standardized coding of specific features by one or more rater. Some studies have used such systems on images of individuals with CFM obtained using 2- and 3-dimensional imaging [14–16]. However, to our knowledge, a comparison of classification based on facial photographs to those carried out through an in-person physical examination has not been published.

The purpose of the current study is to measure the reliability of classification of the common facial features associated with CFM based on an in-person facial examination compared with an assessment based on a standardized set of two-dimensional images.

## Methods

### Data collection

**Fig. 1:**
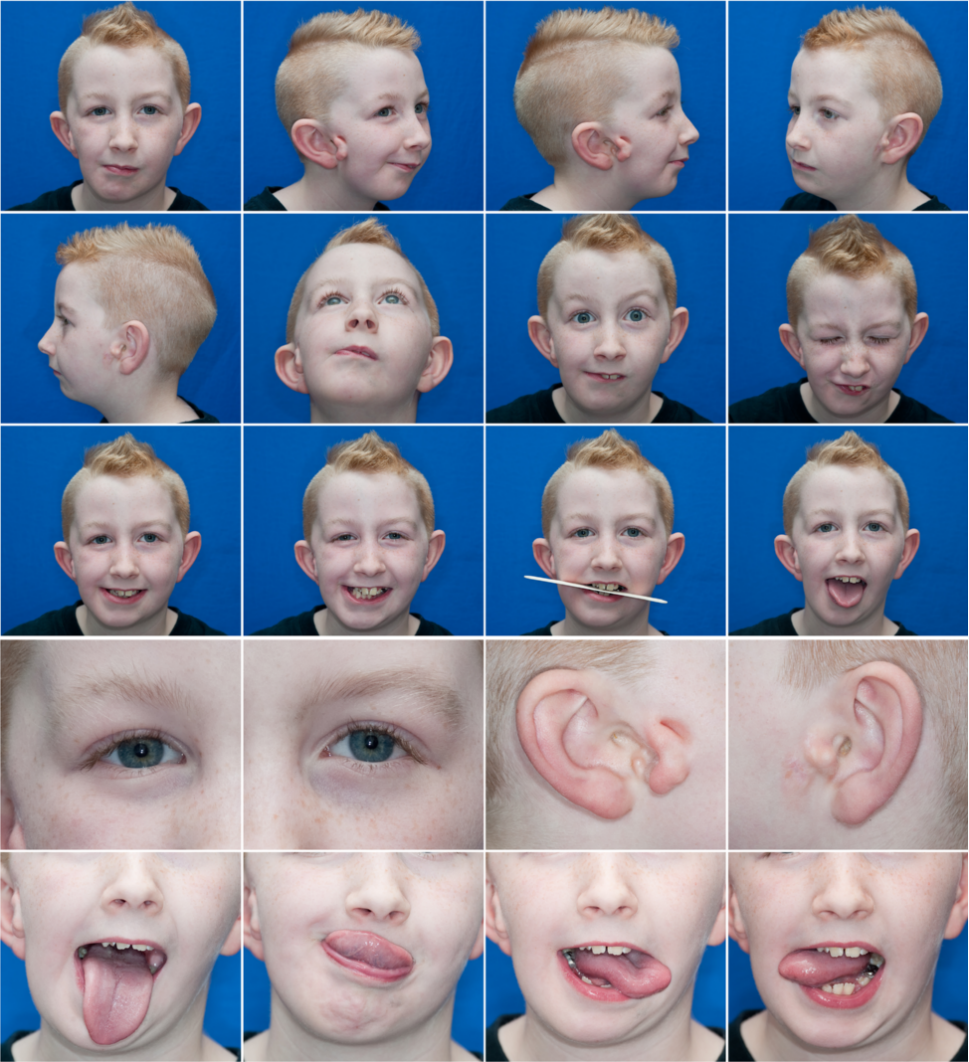
Photographic protocol for individuals with CFM. An example of a study visit contact sheet generated following the previously published protocol [16]

We enrolled children ages 0–21 years who met the research eligibility criteria previously established by the Facial Asymmetry for Interdisciplinary Assessment and Learning (FACIAL) network (Table 1) and were consecutively evaluated in 2014 at a single tertiary care craniofacial center. Study procedures included an interview to collect demographic and clinical history, 16 standardized photos [16] (Fig. 1) and in-person facial exam.

**Table 1:** Eligibility criteria for study participants

Inclusion criteria	
• Younger than 21 years of age	
• At least one of the following:	
1. Microtia	
2. Anotia	
3. Facial asymmetry + Preauricular tag	
4. Facial asymmetry + Facial tag	
5. Facial asymmetry + Epibulbar dermoid	
6. Facial asymmetry + Lateral oral cleft	
7. Preauricular tag + Epibulbar dermoid	
8. Preauricular tag + Lateral oral cleft	
9. Facial tag + Epibulbar dermoid	
10. Lateral cleft + Epibulbar dermoid	
Exclusion criteria	
• Known syndrome	
• Abnormal genetic studies

**Fig. 2:**
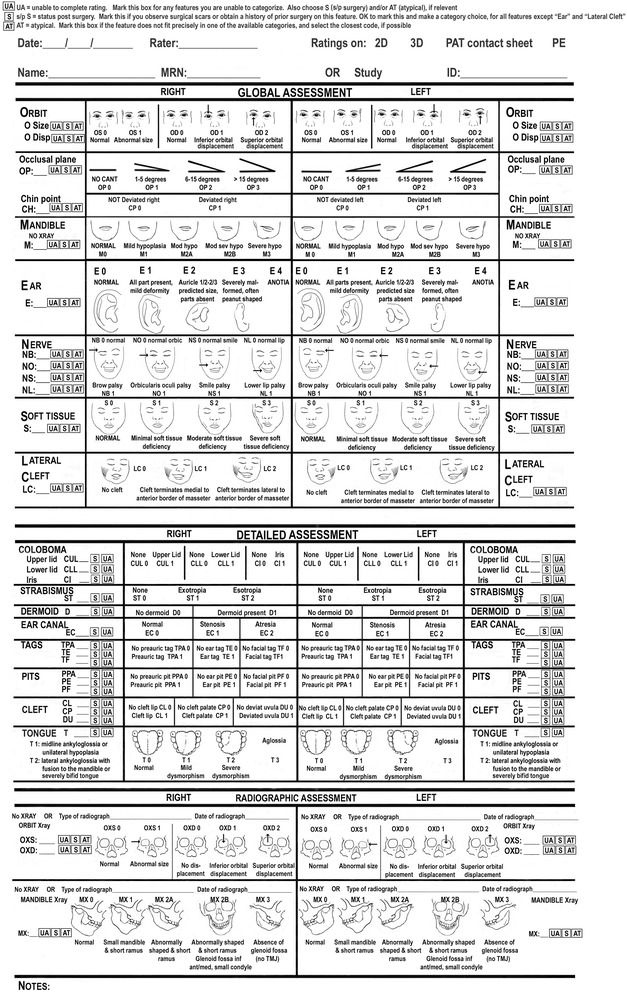
Classification form used in this study based on the modified pictorial OMENS form. Illustrations by Dr. David Low

In-person exams and analysis of photos were sequentially completed by at least one of three clinicians [two pediatricians (CH, KE), and a plastic and reconstructive surgeon (CB)], each of whom has over 5 years experience providing craniofacial care. Clinicians were asked to rate features on each side of the face. In-person exams were performed during the clinic visits, and clinicians recorded the phenotype directly onto an enhanced paper pictorial OMENS data collection form [17] (Fig. 2). Additional features were added and modifications were made in order to increase the likelihood of complete data collection (image not shown).

The photographic assessment was performed at least 30 days following the in-person evaluation, using the 16 standardized photos taken on the day of the in-person exam.

Our photographer cropped the photos and created high resolution PDF files (Fig. 1) that included the standardized views and seven additional images to allow for quick assessment of detail of the eyes, ears, and tongue according to our previously published protocol [16]. Raters viewed the contact sheets on computers with large monitors and were able to zoom in as needed to assess each feature. Clinicians completed ratings on photographs for each individual they examined in person and used the same OMENS data collection template, which included all variables present in the OMENS form, except for cleft palate and bifid uvula as our photo protocol did not include intraoral images. In addition to phenotypic ratings, clinicians were asked to provide descriptions of the subjective experience collecting these data through both modalities.

All data were entered into an Access database by the same individual. All study procedures were approved by our Institutional Review Board (IRB Study #: 14853).

### Data analysis

Descriptive statistics were generated using Stata® (StataCorp, LP College Station, TX, USA).

We used a dataset that combined the 39 left and 39 right sides to create 78 possible ratings for each feature. In addition, we combined data for some of the features related to the same pathological process, such as (1) any tags, (2) any pits, (3) eyelid or iris coloboma, and (4) any facial nerve palsy. We also combined the categories of soft tissue deficiency and mandibular deficiency given the inherent challenges of distinguishing between the causes of lower facial deficiency on photos.

The intraclass correlation coefficient (ICC) was used to measure correlation between ratings of individual features based on the physical exam and the photographic evaluation. ICCs were estimated by fitting random effects models using the ‘lmer’ function in R, with random effects for subject and for side (left or right) as a nested factor within subject. A main effect for side was not included because there was no indication of systematic differences between results for left and right sides. For combined features related to a common pathological process, a single model was fit to the combined data with a random effect to account for the individual features as well as side (left or right). The ICC estimate was calculated as the variance attributed to the random effects divided by the total variance (which also includes the error variance). A 95 % confidence interval for the ICC was calculated using the jackknifed estimate of the standard error. For some features, meaningful ICC estimates and/or confidence intervals could not be obtained because of an insufficient number of cases with positive findings.

We dichotomized the degrees of lower facial hypoplasia by establishing a cutoff for “affected” at a rating of two or greater (on a scale between 0 and 4) for either soft tissue and/or mandibular deficiency. Similarly, we considered a rating of zero or one of the occlusal plane or ear to be “unaffected” and ratings of two or greater were considered to be “affected”.

In order to account for the impact of prior surgery on the ability of raters to accurately identify features which may no longer reflect their presurgical state (e.g. mandibular advancement) or no longer be present (e.g. preauricular tags), raters were instructed to indicate “history of surgery” for any feature for which a surgical incision was identified in the region. If the rater could determine that the incision was directly related to a specific feature, such as a scar in the precise location where a participant likely had lateral cleft, then the rater would indicate “presence of lateral cleft, status post surgery”. However, raters were asked to record “unable to rate, status post surgery” for participants who had undergone ear reconstruction. In these cases, the pre-surgical type of microtia was recoded as “affected” to be greater than or equal to two.

## Results

### Participant characteristics

Thirty-nine participants were enrolled into the study. The average age was 8.4 years (range 3 months-21 years), and approximately 33 % of children were less than 6 years of age. Most participants were white (46 %) and non-Hispanic (82 %). Participants had a wide range of clinical variability, which included: isolated microtia or anotia (*n* = 8), facial asymmetry with microtia (*n* = 15), facial asymmetry without microtia but with other features from the FACIAL inclusion criteria (*n* = 5); or at least two other features from the FACIAL inclusion criteria without facial asymmetry or microtia (*n* = 5) (Table 2).

**Table 2:** Select demographic and phenotypic characteristics of 39 participants with CFM based on physical exam^a^

	All (*n* = 39)	Microtia/anotia only (*n* = 8)	Facial hypoplasia and microtia^b^ (*n* = 15)	Facial hypoplasia without microtia^c^ (*n* = 5)	Microtia without facial hypoplasia^b^ (*n* = 6)	Neither facial hypoplasia nor microtia^c^ (*n* = 5)
Characteristic	*n* (%)	*n* (%)	*n* (%)	*n* (%)	*n* (%)	*n* (%)
Race						
White	18 (46)	3 (38)	8 (50)	4 (80)	3 (60)	0 (0)
Asian	14 (36)	2 (25)	6 (38)	1 (20)	2 (40)	3 (60)
Native American	2 (5)	2 (25)	0 (0)	0 (0)	0 (0)	0 (0)
Other	5 (13)	1 (13)	2 (13)	0 (0)	0 (0)	2 (40)
Ethnicity						
Non-Hispanic	32 (82)	6 (75)	13 (81)	5 (100)	4 (80)	4 (80)
Hispanic	7 (18)	2 (25)	3 (19)	0 (0)	1 (20)	1 (20)
Age						
<1	2 (5)	0 (0)	0 (0)	0 (0)	1 (17)	1 (20)
1–5	11 (28)	2 (25)	5 (33)	2 (40)	2 (33)	0 (0)
>5–10	13 (33)	2 (25)	5 (33)	1 (20)	3 (50)	2 (40)
>10–15	5 (13)	1 (13)	1 (7)	1 (20)	0 (0)	2 (40)
>15–21	8 (21)	3 (38)	4 (27)	1 (20)	0 (0)	0 (0)
Sex						
Male	20 (51)	4 (50)	7 (47)	5 (100)	2 (33)	2 (40)
Microtia or Anotia						
None	10 (26)	0 (0)	0 (0)	5 (100)	0 (0)	5 (100)
Unilateral	26 (67)	8 (100)	12 (80)	0 (0)	6 (100)	0 (0)
Bilateral	3 (8)	0 (0)	3 (20)	0 (0)	0 (0)	0 (0)
Facial Hypoplasia						
None	19 (49)	8 (100)	0 (0)	0 (0)	6 (100)	5 (100)
Unilateral	17 (44)	0 (0)	14 (93)	3 (60)	0 (0)	0 (0)
Bilateral	3 (8)	0 (0)	1 (7)	2 (40)	0 (0)	0 (0)
Dermoid						
None	36 (92)	8 (100)	14 (93)	5 (100)	5 (83)	4 (80)
Unilateral	3 (8)	0 (0)	1 (7)	0 (0)	1 (17)	1 (20)
Any Tag						
None	21 (54)	8 (100)	9 (60)	1 (20)	1 (17)	2 (40)
Unilateral	14 (36)	0 (0)	5 (33)	3 (60)	3 (50.0)	3 (60)
Bilateral	4 (10)	0 (0)	1 (7)	1 (20)	2 (33)	0 (0)
Any Pit						
None	36 (92)	8 (100)	12 (80)	5 (100)	6 (100)	5 (100)
Unilateral	3 (8)	0 (0)	3 (20)	0 (0)	0 (0)	0 (0)
Lateral Oral Cleft						
None	33 (85)	8 (100)	12 (80)	3 (60)	5 (83)	5 (100)
Unilateral	5 (13)	0 (0)	3 (20)	1 (20)	1 (17)	0 (0)
Bilateral	1 (3)	0 (0)	0 (0)	1 (20)	0 (0)	0 (0)

^a^Each clinician rated at least 11 participants (*n* = 11, 15 and 13, respectively)

^b^With or without other features from the FACIAL inclusion criteria

^c^With other features from the FACIAL inclusion criteria

### Clinician subjective impressions

Overall, the raters found the 1-page format of the OMENS form to be useful and efficient for both the in-person and photo evaluations.

For the physical exam rating, raters noted that the length of time and attention to detail required to complete study ratings using the OMENS data collection form was greater than the time typically required for a clinical exam. Clinicians also noted that the recording of some features, such as the presence of small pits, were often not directly relevant to patient care, and added to the burden of physical examination for the provider and participant. They noted that it was difficult to fully assess all features in young, mobile toddlers, particularly those related to symmetry and nerve function. Raters commented on the difficulty ensuring that all data was collected before exiting the exam room; and frequently returned to complete missing data. They reported greater ease of assessment of soft tissue deficiency, mandibular hypoplasia, and ear canal patency with in person exam as compared to photos. Surgical scars were also easier to assess in person, particularly when combined with a parent and/or participant interview at the time of the exam.

The raters found the photos easy to assess when the acquisition protocol was followed. However, for younger children who were often not able to comply with the photo protocol, clinicians noted that many features could not be assessed on the incomplete image set. For example, children for whom an adequate frontal photo was not available routinely have missing data for mandible, soft tissue, and orbital placement.

### Descriptive characteristics of the photographic and in-person ratings

Table 3 includes the distribution of phenotypic characteristics among all participants, as designated both from in-person physical exam as well as photographic ratings. Photographic ratings were more likely to have missing ratings for the tongue: 12 vs. 2, and cleft lip: 6 vs 0, for photographic and PE, respectively; however, orbital displacement was more likely to be missing on in-person ratings than for photographic ratings (5 vs. 0 missing ratings, respectively). Nerve palsies were more likely to be rated as present during in person exams and unable to rate on the photographic assessments. There was not a clear pattern of either type of rating, physical exam or photograph, being more likely to designate a feature as abnormal. The rates of surgery were very low for most features (less than 5 %). Features with the highest surgical rates included: tags (28 %) and ear (6 %) (Table 3).

**Table 3:** Distribution of ratings based on physical exam (PE) and 2D images for 78 hemifaces of 39 participants

	2D photo (*n* = 78)	PE (*n* = 78)		2D photo (*n* = 78)	PE (*n* = 78)		2D photo (*n* = 78)	PE (*n* = 78)
Craniofacial feature	*n* (%)	*n* (%)	Craniofacial feature	*n* (%)	*n* (%)	Craniofacial feature	*n* (%)	*n* (%)
Orbital size			Brow palsy			Ear		
Normal	77 (99)	75 (96)	None	60 (97)	65 (90)	Unaffected		
Abnormal	1 (1)	3 (4)	Present	2 (3)	7 (10)	Normal	35 (46)	36 (47)
*Missing*	*0*	*0*	*Missing*	*16*	*6*	All parts present, mild	11 (15)	9 (12)
History of surgery	none	none	History of surgery	none	none	deformity		
Orbital displacement			Orbic palsy			Affected		
Normal	63 (81)	62 (85)	None	68 (100)	69 (96)	Auricle 1/2-1/3 of	6 (8)	7 (9)
Inferior displacement	6 (8)	6 (8)	Present	0 (0)	3 (4)	predicted size, not		
Superior displacement	9 (12)	5 (7)	*Missing*	*10*	*6*	all parts present		
*Missing*	*0*	*5*	History of surgery	none	none	Severely malformed^c^	22 (29)	24 (31)
History of surgery	none	none	Smile palsy			Anotia	2 (3)	1 (1)
Occlusal plane			None	69 (100)	77 (99)	*Missing*	*2*	*1*
Unaffected			Present	0 (0)	1 (1)	History of surgery^b^	5 (6)	4 (5)
No cant	44 (70)	45 (68)	*Missing*	*9*	*0*	Ear canal		
1–5°	11 (18)	9 (14)	History of surgery	none	none	Normal	35 (53)	44 (60)
Affected			Lower lip palsy			Stenosis	3 (5)	6 (8)
6–15°	7 (11)	11 (17)	None	56 (85)	63 (83)	Atresia	28 (42)	24 (32)
>15°	1 (2)	1 (2)	Present	10 (15)	13 (17)	*Missing*	*12*	*4*
*Missing*	*15*	*12*	*Missing*	*12*	*2*	History of surgery	1 (1)	1 (1)
History of surgery	none	none	History of surgery	none	none			
Mandible						Preauricular tag		
Unaffected			Upper lid coloboma			None	58 (78)	61 (86)
Normal	42 (54)	39 (50)	None	75 (96)	76 (97)	Present	16 (21)	10 (14)
Mild hypoplasia	15 (19)	14 (18)	Present	3 (4)	2 (3)	*Missing*	*4*	*7*
Affected			*Missing*	*0*	*0*	History of surgery	8 (10)	9 (16)
Moderate hypoplasia	9 (12)	8 (10)	History of surgery	none	none	Ear tag		
Mod-sev hypoplasia	7 (9)	11 (14)	Lower lid coloboma			None	69 (92)	67 (94)
Severe hypoplasia	5 (6)	6 (8)	None	78 (100)	78 (100)	Present	6 (8)	4 (6)
*Missing*	*0*	*0*	History of surgery	none	none	*Missing*	*3*	*7*
History of surgery	0 (0)	3 (3.8)	Iris coloboma			History of surgery	6 (8)	7 (9)
Soft tissue deficiency			None	72 (100)	78 (100)	Facial tag		
Unaffected			*Missing*	*6*	*0*	None	71 (96)	70 (97)
Normal	38 (49)	38 (49)	History of surgery	none	none	Present	3 (4)	2 (3)
Minimal	23 (30)	20 (26)	Strabismus			*Missing*	*4*	*6*
Affected			None	75 (97)	77 (100)	History of surgery	8 (10)	7 (9)
Moderate	15 (20)	17 (22)	Exotropia	2 (3)	0 (0)	Preauricular pit		
Severe	1 (1)	3 (4)	*Missing*	*1*	*0*	None	74 (99)	74 (97)
*Missing*	*1*	*0*	History of surgery	none	none	Present	1 (1)	2 (3)
History of surgery	0 (0)	1 (1)	Dermoid			*Missing*	*3*	*2*
Cleft lip			None	75 (96)	74 (95)	History of surgery	3 (4)	2 (3)
None	71 (99)	75 (96)	Present	3 (4)	4 (5)	Ear pit		
Present	1 (1)	3 (4)	*Missing*	*0*	*0*	None	73 (97)	74 (97)
*Missing*	*6*	*0*	History of surgery	0 (0)	1 (1)	Present	2 (3)	2 (3)
History of surgery	none	none	Lateral oral cleft			*Missing*	*3*	*2*
Tongue			No cleft	71 (92)	70 (91)	History of surgery	3 (4)	2 (3)
Normal	62 (94)	74 (97)	Cleft terminates medial^a^	6 (8)	4 (5)	Facial pit		
Mild dysmorphism	4 (6)	2 (3)	Cleft terminates lateral^a^	0 (0)	3 (4)	None	74 (100)	77 (100)
*Missing*	*12*	*2*	*Missing*	*1*	*1*	*Missing*	*4*	*1*
History of surgery	none	none	History of surgery	3 (4)	3 (4)	History of surgery	4 (5)	1 (1)

^a^to anterior border of masseter

^b^non-missing ear ratings which also had a history of surgery corresponded to ratings of “severly malformed, often peanut shaped” and “anotia”; these ear ratings were carried out despite history of surgery since surgery did not affect raters ability to judge severity of ear involvement

^c^often peanut shaped

*Hx* history

### Reliability of phenotypic classification

The percent agreement between ratings on photographs and physical exam was greater than 80 % for all 15 categories included in the analysis. The ICCs were higher than 0.6 for most features; although the confidence intervals were wide. Features with the highest ICC’s included: dermoid, ear abnormalities, and colobomas (ICC’s 0.85, 0.81, and 0.80, respectively). Orbital size, presence of pits, tongue abnormalities, and strabismus had the lowest ICCs, with values less than 0.17. The percent agreement for surgical history by feature was greater than 90 % for most categories (Table 4).

**Table 4:** Prevalence and percentage agreement between physical exam and 2D image ratings, intraclass correlation coefficient (ICC) estimates, and agreement on surgery history for each facial feature. Features are listed in decreasing order of the estimated ICC

Craniofacial feature	Prevalence by PE ratings: proportion (%)^a^	Prevalence by 2D ratings: proportion (%)^a^	Agreement between PE and 2D ratings: proportion (%)	Intraclass correlation coefficient (95 % CI)	Agreement on surgery history: (%)
Dermoid	4/78 (5 %)	3/78 (4 %)	77/78, (99 %)	0.85 (0.51, 1)	99 %
Ear	35/77 (46 %)	36/76 (47 %)	70/76, 92 %)	0.81 (0.51, 1)	99 %
Coloboma	2/234 (1 %)	3/228 (1 %)	227/228, (99 %)	0.80 (0.32, 1)	100 %
Ear canal	24/75 (32 %)	28/65 (43 %)	54/62, (87 %)	0.73 (0.56, 0.89)	97 %
Occlusal plane	12/66 (18 %)	8/63 (13 %)	56/61, (92 %)	0.69 (0.35, 1)	100 %
Mandible & Soft Tissue	46/156 (30 %)	36/155 (23 %)	135/155, (87 %)	0.67 (0.48, 0.86)	97 %
Palsy	24/300 (8 %)	12/263 (5 %)	250/261, (96 %)	0.65 (0.35, 0.95)	100 %
Tags	15/214 (7 %)	26/223 (12 %)	196/210, (93 %)	0.61 (0.39, 0.83)	90 %
Lateral Oral Cleft	7/77 (9 %)	6/77 (8 %)	72/77, (94 %)	0.58 (0.13, 1)	97 %
Cleft lip	3/78 (4 %)	1/72 (1 %)	70/72, (97 %)	0.48 (0, 1)	100 %
Orbital displacement	10/73 (14 %)	16/78 (20 %)	60/73, (82 %)	0.38 (0, 0.81)	100 %
Strabismus	0/77 (0 %)	2/77 (3 %)	75/76, (99 %)	0.17 (0, 1)	100 %
Tongue	2/78 (3 %)	4/64 (6 %)	58/64, (91 %)	0.07 (0, 0.32)	100 %
Pits	4/229 (2 %)	3/224 (1 %)	215/222, (97 %)	0.07 (0, 0.23)	96 %
Orbital size	3/78 (4 %)	1/78 (1 %)	74/78, (95 %)	0 (NA)	100 %

^a^n/N (%): where N is number of ratings, n is number of positive ratings, and the percentage of positive ratings is listed in the brackets

Analysis by age less than or greater than 6 years showed similar results for the percent agreement and ICCs between the two subgroups. Ear canal was the only feature for which a meaningful difference was observed (data not shown). Upon further review of the underlying data, most discrepancies were attributable to misclassification with atresia rated as ‘unaffected’ on physical exam ratings, and the lack of concordance did not seem to be related to an effect of age.

## Discussion

Successful multi-center clinical research in depends on reliable classification of study data. Digital photography offers the opportunity to easily share facial phenotype data among centers around the world. But, until now, it has not been clear whether evaluation of the facial features of individuals with CFM based on photographs could replace ratings based on an in-person physical exam, which many clinicians consider to be the gold standard.

The percent agreement between ratings on physical examination and photographs were quite high for all features evaluated in this study. However, the ICC values for some features were relatively low, despite high agreement percentages. This discrepancy between high agreement and low ICC appears to be a result of the relatively low prevalence of many of the anomalies included in this study; for example, if there is only one positive finding for a feature by physical exam in 100 cases and this feature is not identified on photo assessments, then the agreement would be 99 % but the lack of agreement on the one positive case has a large negative influence on the ICC. Some of our ICC estimates were impacted by low prevalence, such as epibulbar dermoids (*n* = 4, based on physical examination). In addition, the ICC is also affected by the number of cases with both types of ratings available. Confidence intervals are also relatively wide in this study because of the limited number of positive findings for some features.

### Advantages of photos for evaluating craniofacial characteristics

Some features were considered to be easier to assess on photos. Our researchers found it easier to accurately classify degrees of microtia using photos compared to in-person exams. It may be that examiners felt more comfortable taking the time to analyze the features in more detail on photos rather than in a clinical setting.

Our study also found advantages of photographic assessment with regards to time limitations encountered in the clinical setting. It is difficult to set aside more than a few minutes for a focused clinical exam in a busy outpatient clinic. These pressures may create a rushed environment and it may be difficult to return to a component of the exam for re-evaluation if the provider was unsure of the phenotypic findings. With photos, on the other hand, these time limitations are lifted and flexibility for accurately completing the ratings is improved. Acquisition of the photos takes only a few minutes of the patient’s time. Most importantly, the provider does not need to be present. Once the photos are acquired, the reviewer can take as much time as needed to accurately diagnose the phenotype, and do so without the pressures inherent in the clinical environment.

Additionally, phenotypic assessment from the photographic protocol relinquishes some of the awkwardness that can be inherent in completing a comprehensive standardized phenotype protocol through an in-person evaluation. For instance, it can be uncomfortable for the patient, especially a teenager, to have a provider carefully analyze their facial differences all the while marking down their “anomalies” one by one on a score sheet. This seemed contradictory to our goal of focusing on the child or teen’s function, positive self-perception, and as a clinician rater, this felt contradictory to the goals of our practice.

Clinicians noted that the OMENS data collection form used for the study contained 78 features, many of which required detailed examination for accurate coding. Though physicians typically develop a preference for the order in which they complete the assessment, this often varies to accommodate the patient’s needs during the clinic visit. For example, systematic examinations are much easier to complete in a cooperative teenager than a mobile toddler. However, infants and children seem much more eager to sit for a camera, particularly with the help of an engaged photographer.

Although the form was designed to minimize the amount of missing data, the clinicians noted that it still required attention to detail to ensure all fields were complete. In future studies, we will create an electronic version of the rating form to facilitate real-time identification of missing data fields.

Finally, phenotypic assessment using the photographic protocol allows for centralized rating of images. Digital photos are easily shared amongst researchers at various sites who can remotely access the files from a central repository and eliminates the need to have an examiner at each site.

### Advantages of in-person examination

Despite the aforementioned benefits of the photographic assessment, the in-person examination also has a number of advantages. The clinicians found the in-person examination to be more accurate for evaluating soft tissue deficiency and mandibular hypoplasia. Both features are best appreciated through three dimensional assessment, and palpation. Lighting and shadows can affect the degree of perceived asymmetry based on images and the in-person exam allows the patient to move so the examiner can assess the quality and quantity of tissue from different angles. Moreover, both soft tissue and mandibular asymmetry can be enhanced or mitigated with animation. The in-person examination allows the evaluator to interact with the patient and ask them to move their face or jaw in various ways to better assess the degree of asymmetry.

The photographic protocol allows for assessment of dynamic facial nerve function by capturing a series of images of facial expressions; however, the degree of strength or weakness of each facial nerve branch is optimally classified during an in-person examination. In addition, the presence of synkinesis can be difficult to diagnose on photos alone. For individuals with atypical expressions on the photo protocol, it may be difficult for the rater to differentiate between synkinesis, facial palsy, and lack of adherence to the photo protocol. Augmenting the current photographic protocol with video capture of an individual may improve the assessments of facial asymmetry and facial movement and allow for more complete assessment of CFM features.

Finally, there are aspects of the facial phenotype in CFM that are not captured by the photographic protocol used in this study. We did not attempt to obtain intra-oral images of the soft palate and images with adequate angles to assess the external auditory canal were difficult to obtain. Future studies requiring accurate data on such features could incorporate intraoral or intra-aural photographs into the current photo protocol to improve accuracy of the phenotypic assessment of these areas.

### Limitations

This study focused on the reliability of the OMENS tool, and on specific craniofacial features that would be relevant to multicenter research in CFM. Our goal was to determine whether phenotypic classification based on ratings from facial photos would be reliable for evaluation of cohort differences for future research, and not intended to be used for individual patient care nor surgical planning. We have previously evaluated the reliability of the OMENS rating scale using 2D vs. 3D images [15]. We did not test the reliability of in-person exams due to our desire to limit the burden on the study participants.

Confidence intervals for ICC were wide due to the limited sample size, which was typical for a reliability study. We also recognize that the phenotypic variability is high in CFM and not entirely represented by this cohort. Estimates of ICC could be different in other populations with a higher or lower prevalence of these features and for different age groups, although no clear differences were observed based on this age in this study.

### Future directions

In the next phases of this work, we plan to develop resources to facilitate electronic data capture to allow for real-time identification of missing data and to minimize the likelihood of errors in data entry. We will also continue to optimize the protocol for completing ratings. We have summarized some considerations to enhance the reliability for raters using the modified OMENS form in Table 5. We will also continue to explore various combinations of two-dimensional photos, three-dimensional surface images, video, and physical examination to capture more comprehensive data for specific research questions designed to improve craniofacial care for children with CFM.

**Table 5:** This OMENS form used for this study was adapted from prior studies to enhance usability and optimize complete data collection for clinical studies

This form is focused specifically on the common facial features that are associated with CFM, and does not include extracranial features (e.g. cervical spine anomalies, heart, and kidneys) that can occur frequently in children with CFM. Therefore, this tool is not intended to provide comprehensive phenotypic classification in CFM, but instead it is focused on optimizing complete and reliable data collected for common facial features.	
*Adapted from*	
	Vento, A. R., et al. The O.M.E.N.S. classification of hemifacial microsomia. *The Cleft palate-craniofacial journal.* 28, 68–76; discussion 77, (1991).
	Horgan, J. E., et al..OMENS-Plus: analysis of craniofacial and extracraniofacial anomalies in hemifacial microsomia. The Cleft palate-craniofacial journal: 32, 405–412, (1995).
	Gougoutas AJ, Singh DJ, Low DW, Bartlett SP. Hemifacial microsomia: Clinical features and pictographic representations of the OMENS classification system. Plast Reconstr Surg. 2007;120:112e–120e.)
*Prior publications regarding the use of the revised pictorial OMENS characterization form for facial features:*	
	Birgfeld CB, et al. A phenotypic assessment tool for craniofacial microsomia. Plast Reconstr Surg 2011, 127(1):313–320.
	Birgfeld CB, et al. Comparison of Two-Dimensional and Three-Dimensional Images for Phenotypic Assessment of Craniofacial Microsomia. Cleft Palate Craniofac J 2012.
	Heike CL, et al. Photographic Protocol for Image Acquisition in Craniofacial Microsomia. Head Face Med 2011, 7(1):25.
The multi-view imaging protocol enables raters to use multiple images to assess facial features.	
Orbit	
	Malformations of the orbit in CFM commonly include small size, and/or displacement. The appearance of the orbit may be impacted by multiple factors, such as challenges identifying the midline plane in a child with significant facial asymmetry. This rating scale does not distinguish between degree of variation in size or displacement, and some degree of orbital asymmetry is common in the general population.
Occlusal plane	
	Adequate classification requires use of a tongue blade; however, the appearance of the tongue blade angle in the photos may not accurately reflect the degree of maxillary asymmetry. The ultimate classification should be based on the rater’s interpretation of the most appropriate angle.
	S/p surgery: Mark "yes" if evidence to suggest prior surgery. OK to rate severity on current image, despite history of surgery.
	Unable to rate: Mark this category if the tongue blade is not used or not properly positioned and the rater is unable to reliabilty approximate the symmetry of the maxillae.
Mandible	
	Mandibular asymmetry is a hallmark of CFM and is classically attributable to hypoplasia of the ramus. Mandibular hypoplasia can be difficult to evaluate on two-dimensional images. Our protocol incorporates multiple views of the mandible to enhance the rater’s ability to characterize the mandible. Mild mandibular asymmetry can be common in the general population.
	S/p surgery: Mark “yes” if evidence to suggest prior mandibular surgery. OK to rate on current image, despite history of surgery
	Unable to rate: if feature is not well-visualized
Ear	
	CFM is frequently associated with various grades of microtia with or without absence of the external auditory meatus. We have incorporated profile, oblique, and frontal views to allow for assessment of ear size, shape, and position. This system relies on assessment of morphology, and does not account for measurements. Must see all parts of the ear to rate.
	S/p surgery: should be used for all instances in which the original appearance of the ear has been modified by surgery. This feature cannot be rated if the ear has been significantly altered surgically.
	Unable to rate: applied to all ears in which features (such as the helix) are obscured by hair, or the feature has been signicantly altered surgically
Nerve	
	Facial palsies can involve any or all branches of the facial nerve and may be unilateral or bilateral. The photographic protocol includes series of images designed to capture the participant in a neutral expression, and well as animation (following instructions by the photographer) that requires function of each branch of the facial nerve. It can be challenging on images to distinguish between asymmetric movement related to nerve function, and asymmetry that occurs as a result of the underlying structural malformations. Movement does not have to be symmetric to be considered functional. Given the challenges identifying nerve function on a series of static images, we classify “present” if images suggest true paralysis.
	S/p surgery: typically unable to determine based on images
	Unable to rate: Low threshold for not rating if inadequate images of animation are obtained
Soft Tissue	
	Deficiency of the soft tissue is common in CFM. As described for the mandible, capturing soft tissue deficiency and the resultant facial asymmetry can be challenging using a 2D images. For this reason, we’ve included several views of the face to allow for assessment of soft tissue asymmetry.
	S/p surgery: Any evidence of scarring that could indicate prior surgery affecting this feature; OK to rate feature with or without evidence of prior surgery
	Unable to rate: if feature is not well-visualized
Lateral Cleft (Macrostomia)	
	S/p surgery: OK to rate degree based on the location of the scar
	Unable to rate: inadequate information to complete the rating with confidence, based on poor image or scarring that is not clearly related to prior cleft repair
Coloboma	
	S/p surgery: often challenging to identify on images
	Unable to rate: Inadequate information to complete the rating with confidence
Strabismus	
	Typically based on the identification of an asymmetric corneal light reflex
	S/p surgery: Not applicable to ratings based on photos
	Unable to rate: Can be difficult to assess on photos
Dermoid	
	Can be difficult to assess on photos, particularly if eyes are not fully open and/or the dermoids have been treated surgically. Artifact on images is also common and may interfere with an accurate assessment
	S/p surgery: often challenging to identify on images
	Unable to rate: Inadequate information to complete the rating with confidence
Ear Canal	
	Can be challenging to obtain an adequate view of the ear canal on photos. Raters cannot identify ear canal stenosis on photos
	S/p surgery: Mark this category if evidence of surgical ear/canal reconstruction
	Unable to rate: Mark this category if the rater is unable to determine with certainty the presurgical appearance of the canal
Tags and Pits	
	S/p surgery: Mark this category if the rater identifys scars suggesting prior ear or soft tissue surgery in the location of preauricular or facial tags. If the surgical scars are characteristic for preauricular or facial tag removal, OK to also mark “present” and “s/p” surgery for these features.
	Unable to rate: if evidence of prior surgery and the rater cannot identify the presurgical state.
Cleft lip	
	S/p surgery: OK to rate degree based on the scar
	Unable to rate: Inadequate information to complete the rating with confidence
Cleft palate. The photographic protocol does not include an intraoral view, currently based only on physical exam.	
	S/p surgery: OK to rate degree based on the appearance of the scar
	Unable to rate: Inadequate information to complete the rating with confidence
Tongue	
	Unable to rate: Mark this category if the views are insufficient to confidently classify the morphology of the tongue
Radiographic features: We have not formally tested the reliability of radiographic illustrations for the orbits and mandible.	
Please contact daniela.luquetti@seattlechildrens.org or carrie.heike@seattlechildrens.org for more detailed questions	

### Consent

Written informed consent was obtained from the participant’s parent for the publication of this report and any accompanying images.

## Conclusion

Use of the Phenotypic Assessment Tool for Craniofacial Microsomia (PAT-CFM), which combines a pictorial OMENS tool and a standardized set of 2-dimensional photographs, is equivalent to in-person exam for most phenotypic features and better for some aspects of assessment of patients with CFM. Thus, the PAT-CFM can be used for multi-center research studies in CFM in lieu of in patient exams.

## Abbreviations

CFM: craniofacial microsomia
FACIAL: facial asymmetry collaborative for interdisciplinary assessment and learning
OMENS: classification system for orbital anomalies in size and position, mandibular hypoplasia, ear malformations (microtia), facial nerve palsy, and facial soft tissue deficiency
